# Эффективность лечения агонистами рецептора глюкагоноподобного пептида-1 азиатских пациентов с сахарным диабетом типа 2

**DOI:** 10.14341/probl13245

**Published:** 2023-05-11

**Authors:** Л. Ю. Хамнуева, Л. С. Андреева

**Affiliations:** Иркутский государственный медицинский университет; Иркутский государственный медицинский университет

**Keywords:** Сахарный диабет, азиатская популяция, глюкагоноподобный пептид-1, агонист рецептора глюкагоноподобного пептида-1, пероральный семаглутид

## Abstract

**ОБОСНОВАНИЕ:**

ОБОСНОВАНИЕ. Прогнозируемый рост числа пациентов с сахарным диабетом типа 2 (СД2) через два десятилетия на 68% и особенности патофизиологического течения заболевания являются важнейшими факторами для выработки оптимальной тактики лечения заболевания у азиатского населения. На сегодняшний день известно, что дисфункция β-клеток является доминирующей в патогенезе СД2 у азиатов. В ряде азиатских стран инкретиннаправленная терапия является ведущей.

**ЦЕЛЬ:**

ЦЕЛЬ. Проведение анализа работ, посвященных изучению особенностей секреции глюкагоноподобного пептида-1 (ГПП-1) и результатов клинических исследований препаратов класса агонистов рецепторов ГПП-1 (арГПП-1), а также оценка их эффективности лечения у азиатского населения с СД2.

**МАТЕРИАЛЫ И МЕТОДЫ:**

МАТЕРИАЛЫ И МЕТОДЫ. Обзор исследований, посвященных патофизиологическим аспектам секреции ГПП-1 и оценке эффективности терапии препаратами арГПП-1, зарегистрированными и применяющимися в реальной клинической практике в азиатских регионах.

**РЕЗУЛЬТАТЫ:**

РЕЗУЛЬТАТЫ. Ряд исследований в азиатских странах показал, что уровни интактного ГПП-1 были значительно ниже как у пациентов с СД2, так и у здоровых добровольцев из Японии; а также на стадии нарушенной толерантности   к глюкозе. Предполагается, что причиной снижения уровня ГПП-1 является, или нарушение его секреции в кишечнике, или ускоренный процессинг дипептидилпептидазой 4-го типа, или их сочетание. Большая эффективность лечения арГПП-1 в достижении гликемического контроля у азиатских пациентов с СД2 представлена Kim Y.G. и соавт.  в метаанализе 15 рандомизированных контролируемых исследований, снижение уровня гликированного гемоглобина (HbA1c) на фоне лечения арГПП-1 в среднем составило -1,16% в исследованиях с преобладанием азиатских пациентов и -0,83% — неазиатских. В клинической программе PIONEER-9 были получены подобные результаты, пероральный семаглутид более выраженно влиял на гликемический контроль у японских пациентов. Так, среднее изменение HbA1c составило в дозе 3 мг -1,1%, 7 мг — -1,5%, 14 мг — -1,7%; тогда как в исследовании PIONEER-1 в глобальной популяции среднее изменение HbA1c составило при лечении в дозе 3 мг -0,6%, в дозе 7 мг — -0,9% и в дозе 14 мг — -1,1%. Исследование PIONEER-10 позволило сделать выводы о хорошей переносимости перорального семаглутида японскими пациентами с СД2. Пероральный семаглутид снижал уровень HbA1c (доза 14 мг) и массу тела (дозы 7 и 14 мг) более значимо в сравнении с дулаглутидом в дозе 0,75 мг. Результаты объединенного анализа оценки арГПП-1 длительного действия показали более существенное снижение риска сердечно-сосудистых событий в азиатской субпопуляции.

**ЗАКЛЮЧЕНИЕ:**

ЗАКЛЮЧЕНИЕ. Представленные результаты исследований позволяют предположить преимущества в выраженности гликемического контроля, а также в снижении относительных рисков сердечно-сосудистых событий при лечении арГПП-1 в азиатской популяции, что требует дальнейших углубленных исследований и имеет значение для оптимальной тактики ведения пациентов с СД2.

## Abstract (en)

**BACKGROUND:**

BACKGROUND. The projected 68% increase in patients with type 2 diabetes mellitus (T2D) in the upcoming decades and the specific pathophysiological course of the disease are critical factors for the development of optimal disease management tactics in the Asian population. It is now known that β-cell dysfunction is dominant in the pathogenesis of T2D in Asians. In a number of Asian countries, incretin therapy is the leading therapy.

**AIM:**

AIM. To review literature on glucagon-like peptide-1 (GLP-1) secretion and clinical trial results of GLP-1 receptor agonist class (GLP-1RA) drugs as well as to evaluate their effectiveness in Asian population with T2D.

**MATERIALS AND METHODS:**

MATERIALS AND METHODS. A review of studies on pathophysiological aspects of GLP-1 secretion and evaluation of the efficacy of therapy with GLP-1RA preparations registered and used in clinical practice in Asian regions.

**RESULTS:**

RESULTS. Several studies in Asian countries have shown that intact GLP-1 levels were significantly lower in both T2D patients and healthy Japanese volunteers; as well as in patients with impaired glucose tolerance. It is suggested that either impaired secretion of GLP-1 in the gut, accelerated processing by dipeptidyl peptidase-4, or a combination of both are responsible for the decrease in GLP-1. The greater efficacy of GLP-1RA treatment in achieving glycemic control in Asian T2D patients was presented by Kim Y.G. et al. in a meta-analysis of 15 randomised controlled trials, the reduction in HbA1c on GLP-1RA treatment averaged -1.16% in Asian-dominated studies and -0.83% in non-Asian-dominated studies. In the PIONEER 9 clinical programme, similar results were obtained, with oral semaglutide having a more pronounced effect on glycaemic control in Japanese patients. Thus, the mean change in HbA 1c was -1.1%, 7 mg -1.5%, and 14 mg -1.7% at the 3 mg dose; whereas in the PIONEER 1 study in the global population, the mean change in HbA1c was -0.6%, -0.9% and -1.1% for 3, 7, 14 mg semaglutide, respectively. The PIONEER 10 study concluded that oral semaglutide was well tolerated by Japanese patients with T2D. Oral semaglutide reduced HbA1c (14 mg dose) and body weight (7 and 14 mg doses) more significantly compared to dulaglutide at 0.75 mg dose. Results of a pooled analysis of long-acting GLP-1RA showed a more significant reduction in cardiovascular event risk in the Asian subpopulation.

**CONCLUSION:**

CONCLUSION. The presented review describes benefits in glycemic control as well as in the reduction of relative cardiovascular event risks with GLP-1RA treatment in the Asian population, which requires further in-depth research and implies optimal management tactics in patients with T2DM.

Сахарный диабет (СД) относится к категории социально значимых неинфекционных заболеваний; распространенность СД отличается эпидемическими темпами роста. По последним данным Международной федерации диабета (International Diabetes Federation, IDF), во всем мире количество пациентов с СД достигает 537 млн человек, из которых около половины проживают в Азии [1]. Ожидается, что к 2045 г. в мире количество пациентов с СД возрастет на 46%, в том числе в регионе Юго-Восточной Азии предполагаемый прирост составит 68%, в регионе Средней Азии и Северной Африки — 87% [1]. Тенденция к росту случаев СД за последние 20 лет отмечается во многих азиатских странах. Абсолютное большинство публикаций о клиническом течении, патогенетических особенностях СД 2 типа (СД2), результатах проведенных исследований среди азиатского населения представлено авторами из Восточной Азии (Китай, Япония, Корея). Клинические характеристики пациентов с СД2 из Восточной Азии отличаются от таковых у пациентов европейского происхождения; так, у них СД2 развивается при более низком индексе массы тела и в более молодом возрасте [2]. Возникает необходимость изучения факторов, лежащих в основе этих межэтнических различий. Следует отметить, что СД2 у пациентов из Восточной Азии характеризуется в первую очередь постпрандиальной дисфункцией β-клеток [3–5]. У пациентов с СД2 этого региона отмечаются более низкие показатели инсулиногенного ответа β-клеток и инсулинорезистентности [3][6][7]. Поэтому патофизиологические различия в проявлении заболевания имеют решающее значение для соответствующих профилактических и терапевтических подходов. Ранее, из-за сниженной функции β-клеток, средства, стимулирующие секрецию инсулина, — секретагоги, сульфонилмочевина и глиниды использовались в качестве предпочтительных препаратов для лечения СД2 у населения Японии и других народов Восточной Азии. В последнее время терапия на основе инкретинов — ингибиторов дипептидилпептидазы-4 (иДПП-4) и агонистов рецептора глюкагоноподобного пептида-1 (арГПП-1) стала широкодоступной для лечения СД2. Эти препараты инкретиннаправленной терапии улучшают функцию β-клеток за счет глюкозозависимого механизма действия, в связи с чем нивелируются такие риски нежелательных явлений, как гипогликемия или набор массы тела, свойственные ранее упомянутым секретагогам [8]. В регионе Восточной Азии уже к 2015 г. более 70% всех пациентов с СД2 получали иДПП-4 или арГПП-1; из них около 60% не принимали лекарственные препараты ранее, т.е. препараты этой группы были назначены как первая линия сахароснижающей терапии [9].

При проведении исследований действия инкретиновых гормонов для изучения их секреции и процессинга in vivo важно измерять уровень не только интактных (ответственных за эндокринное действие), но и общих (сумма интактных и расщепленных при помощи ДПП-4) форм [10][11]. Показано, что азиаты, особенно выходцы из Восточной Азии, как правило, демонстрируют более низкие уровни интактного ГПП-1, независимо от того, здоровы они или имеют СД2 [12][13]. Предполагается, что низкие уровни интактного ГПП-1 можно объяснить или нарушением его секреции из кишечника, или ускоренным процессингом ДПП-4, или сочетанным действием этих факторов. Поскольку, с одной стороны, пиковые концентрации ГПП-1 адекватно возрастают в ответ на стимуляцию глюкозой у исследуемых, в то время как уровень интактного ГПП-1 остается низким, что может свидетельствовать об усиленной активности ДПП-4. С другой стороны, существует зависимость секреции ГПП-1 от объема пищи и ее состава, в связи с чем возможна роль диетических особенностей в отношении описанного феномена у представителей азиатской популяции [13–15]. Например, систематический обзор (2012 г.) показал, что потребление белого риса несколько раз в неделю увеличивало риск развития СД2 в западных странах на 12%, в то время как среди представителей азиатской популяции, для которых рис является неотъемлемым компонентом ежедневного рациона, ассоциированный риск развития СД2 был значительно выше — 55% [16]. Эти результаты согласуются с другими исследованиями, которые показали, что у азиатов гликемический ответ на одни и те же продукты был выше, чем у представителей европеоидной расы [17–19].

Вызывает интерес исследование пациентов этнической группы «хань» из Китая. По результатам орального глюкозотолерантного теста (ОГТТ) были выделены группы с изолированной нарушенной гликемией натощак (НГН, n=98), нарушенной толерантностью к глюкозе (НТГ, n=101), НГН+НТГ (n=104) и впервые диагностированным СД (n=105), контрольную группу составили пациенты без СД (n=123) соответствующего возраста и пола. Результаты исследования показали снижение общего ГПП-1 натощак в группах НГН+НТГ и впервые диагностированного СД, более чем в 2 раза, в сравнении с контрольной группой (р<0,005). После нагрузки 75 г глюкозы через 2 ч общие концентрации ГПП-1 были значимо ниже в группах НГН+НТГ (22,24±16,7 пмоль/л) и впервые диагностированного СД (16,49±14,11 пмоль/л), по сравнению с контрольной группой (35,39±15,40 пмоль/л, р<0,005). Ценность обсуждаемого исследования возрастает в связи с тем, что пациенты не получали какой-либо сахароснижающей терапии, которая могла бы изменить уровни ГПП-1. Показано, что у лиц с НГН+НТГ и впервые диагностированным СД уже регистрировалось значительное нарушение секреции ГПП-1, которое сопровождалось значительной гипергликемией через 2 ч после приема глюкозы. Исследователи считают, что полученные ими результаты предоставляют ценную информацию о возможностях профилактики и лечения китайских пациентов с СД2 [20]. Похожие результаты получили Yabe D. и соавт., которые показали, что уровень ГПП-1 как натощак, так и после стандартного приема пищи был значительно ниже, при этом интактный уровень ГПП-1 натощак был снижен и у больных СД2, и у здоровых японцев [13]. Ожидаемо, что, по данным ряда исследований, терапия на основе ГПП-1 была более эффективной у японских пациентов с СД2, чем у других этнических групп, что свидетельствует о более глубоком дефиците ГПП-1 у японских пациентов с СД2 [21–23].

В контексте имеющихся сведений о нарушенной секреции ГПП-1 у лиц азиатской популяции представляют интерес результаты следующих метаанализов. Так, Kim Y.G. и соавт. опубликовали результаты метаанализа 55 исследований длительностью не менее 12 нед, в котором провели сравнение гипогликемической эффективности ингибиторов ДПП-4 у азиатских и неазиатских пациентов с СД2. Лучшую эффективность иДПП-4 показали в исследованиях с участием ≥50% участников из Азии, среднее снижение HbA1c достигло 0,92%; в исследованиях с <50% азиатских участников среднее снижение HbA1c составило 0,65%. Разница в снижении HbA1c между группами составила 0,26%, p<0,001 (рис. 1).

Вероятность достижения целевого уровня HbA1c <7,0% на фоне применения иДПП-4 в сравнении с контролем была выше в исследованиях с большей представленностью (≥50%) участников из Азии, чем в исследованиях с меньшим числом (<50%) азиатских участников соответственно) [24].

Эти же исследователи провели метаанализ 15 рандомизированных контролируемых исследований длительностью 12 нед и более с преобладанием азиатов (≥50% участников из Азии) и исследований без преобладания азиатов (<50% участников из Азии). Целью исследований явилось сравнение снижения уровня HbA1с, в качестве оценки эффективности аналогов ГПП-1 у азиатов и неазиатов с СД2. Снижение HbA1c на фоне лечения аналогами ГПП-1 в среднем составило -1,16% в азиатских исследованиях и -0,83% — в неазиатских. Разница между группами составила -0,32% (95% ДИ -0,64–-0,01; p=0,04). Относительный риск (ОР) с 95% ДИ достижения целевого уровня HbA1c ≤7,0%, как правило, был выше в исследованиях, в которых преобладали азиатские страны [ОР 5,7 (3,8, 8,7)], чем в исследованиях, в которых преобладали неазиатские страны [ОР 2,8 (2,4, 3,3)]. Изменения массы тела были одинаковыми между двумя группами. Гипогликемия, как правило, чаще встречалась в исследованиях с преобладанием азиатов [ОР 2,8 (2,3, 3,5)], чем в исследованиях, в которых не преобладали азиаты [ОР 1,5 (1,2, 1,8)], но тяжелая гипогликемия была очень редкой в обеих группах [25]. Таким образом, препараты инкретинового ряда (иДПП-4 и арГПП-1) снижали уровень HbA1c в большей степени в исследованиях с преобладанием азиатского населения [25].

В 2019 г. Kang Y.M. и соавт. опубликовали результаты объединенного анализа оценки сердечно-сосудистых эффектов арГПП-1 длительного действия с особым акцентом на азиатскую субпопуляцию [26]. Три многоцентровых рандомизированных двойных слепых плацебо-контролируемых исследования сердечно-сосудистых исходов (CVOT) арГПП-1 длительного действия были включены в комбинированный анализ: исследование лираглутида LEADER [27], исследование семаглутида SUSTAIN-6 [28] и исследование EXSCEL еженедельного приема эксенатида [29]. Во всех трех CVOT первичным исходом было неблагоприятное сердечно-сосудистое событие (MACE), включавшее сердечно-сосудистую смерть, нефатальный инфаркт миокарда и нефатальный инсульт. Существенное снижение риска в сравнении с другими этническими группами наблюдалось у азиатов (ОР, 0,35; 95% ДИ 0,09–1,32); подгрупповой анализ подтвердил существенное различие по эффекту на MACE между представителями различных рас (р<0,001) (табл. 1) [26].

К аналогичным результатам, подтвердившим лучший прогноз в отношении сердечно-сосудистых исходов у азиатов, пришли Lee M.Y. и соавт. в 2021 г. Исследователи провели метаанализ оценок суммарного отношения рисков (HR) в зависимости от расы по результатам CVOT-исследований ингибиторов глюкозо-натриевых транспортеров 2 типа и арГПП-1. В шести исследованиях арГПП-1 с участием 4195 азиатов и 37 530 европеоидов, MACE составил 0,68 (0,53, 0,84) и 0,87 (0,81, 0,94) соответственно (p=0,03). Авторы считают, что у азиатов очевидна более выраженная польза от терапии арГПП-1 по сравнению с европеоидами, и предполагают необходимость проведения большего количества исследований в азиатских странах для дальнейшего изучения и проверки эффективности арГПП-1 (рис. 2) [30].

Несмотря на то что достаточно хорошо изучены механизмы, лежащие в основе кардиопротективного эффекта арГПП-1, остаются неясными причины, объясняющие различия сердечно-сосудистых эффектов в зависимости от расы. Исследователями обсуждаются следующие аспекты: во-первых, потенциальные метаболические последствия, вызванные более низкими общими и стимулированными уровнями ГПП-1 у азиатов, которые могли быть компенсированы введением экзогенного арГПП-1 с оказанием более значимых эффектов ГПП-1 на организм, в том числе на сердечно-сосудистую систему; во-вторых, за счет особенностей стимуляции рецепторов ГПП-1 у азиатских субъектов, что приводит к более выраженной гипогликемической активности. Наблюдаемая у жителей Восточной Азии более выраженная дисфункция β-клеток, генетические факторы, иной состав пищи могут привести и к другому ответу на введение инкретина [31]. Поскольку у азиатов развитие и течение СД2 происходит при меньшей средней массе тела, объем для распределения препаратов также ниже, в связи с чем при сопоставимых дозировках препаратов азиатские пациенты подвергаются воздействию более высокого уровня арГПП-1 в организме. Все вышеупомянутые аспекты могут в какой-то степени объяснить дополнительную сердечно-сосудистую пользу от арГПП-1, однако их фактическая связь с этнической принадлежностью, а также другие возможные механизмы более выраженного снижения частоты сердечно-сосудистых событий на фоне применения арГПП-1 в азиатских популяциях заслуживают дальнейшего изучения и специальных испытаний в конкретных популяциях [26].

Одним из наиболее эффективных современных представителей арГПП-1 является семаглутид. Он имеет 94% гомологии в последовательности с нативным человеческим ГПП-1; три структурных отличия пролонгируют его период полувыведения примерно до 1 нед, не снижая связывания с его рецепторами ГПП-1 [32].

В клинической программе SUSTAIN семаглутид для подкожного введения демонстрировал у пациентов с СД2 устойчивый гликемический контроль и потерю веса по сравнению с различными препаратами [33][34]. В 30-недельном исследовании SUSTAIN China, проведенном в китайской популяции, семаглутид демонстрировал более высокую эффективность в сравнении с ингибитором ДПП-4 — ситаглиптином. В исследование были включены около 70% (605/868) пациентов из Китая, остальные пациенты являлись представителями четырех других азиатских стран, включая Республику Корея. Обе дозы семаглутида (0,5 мг и 1 мг) превосходили ситаглиптин 100 мг в снижении HbA1c и массы тела через 30 нед лечения. Целевого уровня HbA1c <7,0% достигли 71% пациентов, получавших семаглутид в дозе 0,5 мг в неделю и 82% — 1 мг в неделю, по сравнению с 45% на терапии ситаглиптином 100 мг. Большая доля, 35 и 55%, пациентов в группах семаглутида 0,5 и 1,0 мг соответственно достигли клинически значимой потери веса на 5% и более, что является важным фактором в лечении СД2, связанным с множеством благоприятных физиологических эффектов, включая в том числе улучшение гликемического контроля [35]. Семаглутид также представлен в форме для приема внутрь, что делает его первым и пока единственным препаратом пептидной природы, а следовательно, и единственным арГПП-1, применяемым в таблетированной форме. Для защиты семаглутида от ферментативного расщепления и облегчения его всасывания в желудке после перорального приема был использован усилитель абсорбции N- (8-[2-гидроксибензоил]амино)каприлат натрия (SNAC) [36]. SNAC уменьшает всасывание семаглутида за счет повышения рН локальной среды (защита от протеолитической деградации) и использования пассивного трансцеллюлярного пути проникновения через клеточные мембраны (повышение липофильности комплекса) [37].

До разработки семаглутида для перорального применения все арГПП-1 вводили путем инъекции. Основываясь на опыте применения инсулина, можно предполагать, что инъекционный способ введения может препятствовать использованию этих препаратов. Так, наиболее частыми проблемами для 32,6% врачей и 56% пациентов, получающих арГПП-1, было названо «предпочтение ими пероральных лекарственных средств, нежели инъекций» [38, 39]. В 2021 г. Igarashi A. и соавт. представили данные онлайн-опроса среди японских пациентов с СД2 и HbA1c ≥7,0%, получающих пероральные сахароснижающие препараты и не получавших ранее инъекционную терапию. Для проведения исследования были созданы гипотетические профили терапии с использованием имеющихся данных прямых испытаний в Японии перорального приема семаглутида (7 и 14 мг), инъекционного дулаглутида (0,75 мг) и инъекционного лираглутида (0,9 мг). По мнению респондентов, самыми значимыми пунктами лечения были способ и частота введения (49,1%), затем риск тошноты (30,8%), изменение веса (11,3%) и изменение уровня HbA1с (8,8%). Респондентами было отдано предпочтение пероральному семаглутиду 7 и 14 мг: форме семаглутида 7 мг отдавалось предпочтение перед дулаглутидом (91,0% респондентов) и лираглутидом (89,4%); форма семаглутида 14 мг была предпочтительнее дулаглутида (на 88,2%) и лираглутида (на 94,4%). Готовность инициировать лечение также была выше для перорально вводимых препаратов, подобных семаглутиду: 62,4% в дозе 7 мг и 64,0% в дозе 14 мг в сравнении с 13,6% и 11,0% при формах, подобных инъекционным арГПП-1. Авторы пришли к выводу, что японские пациенты с СД2, по-видимому, предпочитают пероральные формы арГПП-1 инъекционным формам арГПП-1, и способ введения, вероятно, является наиболее важным фактором в этом решении [40].

Эти выводы подтверждает исследование качества жизни, связанного со здоровьем (HRQoL), в исследовании PIONEER-10. Оценка проводилась с использованием специфического для Японии опросника качества жизни (DTR-QoL) при терапии СД. Опросник содержит 29 пунктов для оценки влияния лечения диабета на качество жизни HRQoL в четырех разделах: нагрузка на социальную и повседневную деятельность, беспокойство и неудовлетворенность лечением, гипогликемия, удовлетворенность лечением. Результаты показали, что именно пероральное введение семаглутида в дозах 7 и 14 мг улучшило качество жизни пациентов, измеренное с помощью специального японского инструментария DTR-QoL по сравнению с дулаглутидом в дозе 0,75 мг на 52-й неделе. Таким образом, пероральный прием семаглутида может помочь большему числу пациентов с неконтролируемым СД2 получить терапию арГПП-1 за счет преодоления барьеров, связанных с инъекционной терапией [41].

Ранее уже обсуждалось, что патофизиология СД2 в азиатской отличается от таковой в европейской популяции [31], что, по мнению Araki E. и соавт., обуславливает необходимость более углубленного изучения ответа на лечение СД2 у азиатских пациентов. В международную клиническую программу исследования перорального семаглутида — PIONEER-1, -3, -4 и -8 — включались пациенты из Японии. Araki E. и соавт. провели субанализ в отношении результатов лечения пероральным семаглутидом 7 и 14 мг у данных пациентов и представили следующие результаты: снижение уровня HbA1c по сравнению с исходным уровнем у японских пациентов составило от 1,0 до 1,2% и от 1,4 до 1,7%; снижение массы тела составило от 1,0 до 2,7% и от 3,7 до 4,7% соответственно. Снижение уровня HbA1c и массы тела, как правило, было выше, чем в группах сравнения (плацебо, ситаглиптин 100 мг, лираглутид 1,8 мг в дополнение к инсулину). Как и ожидалось, основными нежелательными явлениями были события, связанные с желудочно-кишечным трактом, чаще всего включающие тошноту, диарею и запор. Исследователи пришли к выводу, что пероральный семаглутид эффективен и хорошо переносится японскими пациентами с СД2. В то же время при рассмотрении результатов для японских пациентов следует отметить, что это были гипотез-генерирующие анализы подгрупп глобальных исследований, которые не были исходно определены в дизайне исследования. Исследования не обладали достаточной мощностью, чтобы продемонстрировать статистическое различие в пользу японской популяции, так как было включено относительно небольшое количество японских пациентов (PIONEER-1: по всему миру — 703 пациента и Япония — 116; PIONEER-3: 1864 и 207; PIONEER-4: 711 и 75; PIONEER 8: 731 и 194 соответственно). В этой связи описанные результаты следует рассматривать только как ориентировочные [42].

В исследование PIONEER-10 были включены пациенты из Японии в возрасте 20 лет и старше с неконтролируемым CД2. Они были рандомизированы для приема семаглутида перорально 1 раз в сутки в дозе 3, 7 или 14 мг или дулаглутида подкожно 1 раз в неделю в дозе 0,75 мг в течение 52 нед в качестве дополнения к основному лечению. Дулаглутид назначался в максимально одобренной в Японии дозе 0,75 мг в неделю. Первичной конечной точкой было количество нежелательных явлений, возникших на фоне лечения в течение 57 нед. Вторичные конечные точки включали среднее изменение по сравнению с исходным уровнем HbA1c и массу тела через 52 нед. Было обследовано 492 пациента, 458 из которых были рандомизированы в группу перорального приема семаглутида 3 мг (n=131), 7 мг (n=132) или 14 мг (n=130) или дулаглутида 0,75 мг (n=65). Завершили исследование 98% пациентов. Нежелательные явления возникли у 77% пациентов, принимавших перорально семаглутид в дозе 3 мг, у 80% — семаглутид в дозе 7 мг, у 85% — семаглутид в дозе 14 мг и у 82% пациентов, получавших дулаглутид. Наиболее частыми нежелательными явлениями были инфекции и желудочно-кишечные расстройства. О случаях смерти или тяжелых гипогликемических случаях не сообщалось. Среднее снижение HbA1c от исходного уровня (8,3%) к 52-й неделе достигло: при пероральном приеме семаглутида 3 мг — 0,9%; 7 мг — 1,4%, 14 мг — 1,7% и при приеме дулаглутида — 1,4%; разница в лечении составила 0,3% для семаглутида перорально в дозе 14 мг по сравнению с дулаглутидом 0,75 мг (p=0,0170). Среднее изменение массы тела от исходного уровня (72,1 кг) к 52-й неделе составило: при пероральном приеме семаглутида 3 мг — 0,0 кг, 7 мг — 0,9 кг; 14 мг — 1,6 кг и при приеме дулаглутида масса тела увеличилась на 1,0 кг (разница в лечении составила -2,6 кг для перорального семаглутида 14 мг по сравнению с дулаглутидом; p<0,0001). Результаты проведенного исследования позволили сделать выводы о хорошей переносимости перорального семаглутида японскими пациентами с СД2. Пероральный семаглутид значительно снижал HbA1c (доза 14 мг) и массу тела (дозы 7 и 14 мг) по сравнению с еженедельным подкожным введением 0,75 мг дулаглутида к 52-й неделе (рис. 3) [43].

В этом же 2020 г. были опубликованы результаты исследования PIONEER 9 — 52-недельного рандомизированного контролируемого исследования фазы 2/3а, проведенного в 16 центрах в Японии. Исследование PIONEER-9 было направлено на оценку дозозависимого влияния перорального семаглутида и сравнение эффективности и безопасности перорального семаглутида с плацебо и подкожным арГПП-1 у пациентов с СД2 в Японии. Пациенты были рандомизированы в группу перорального приема семаглутида 3 мг (n=49), 7 мг (n=49) или 14 мг (n=48) или плацебо (n=49) или лираглутида 0,9 мг (n=48). Наибольшие изменения HbA1c от исходного уровня (в среднем 8,2%) наблюдались к 26-й неделе (первичная конечная точка) и были зависимы от получаемых доз перорального семаглутида (в дозе 3 мг среднее изменение HbA1c -1,1%, 7 мг -1,5%, и 14 мг -1,7%; для плацебо -0,1% и для лираглутида 0,9 мг -1,4%). Расчетная разница HbA1c в сравнении с лираглутидом 0,9 мг составили 0,3% (p=0,0799) для перорального приема семаглутида 3 мг; для 7 мг -0,1% (р=0,394) и для 14 мг -0,3 % (p=0,0272). Наиболее частым классом нежелательных явлений при пероральном приеме семаглутида явились назофарингит, а также желудочно-кишечные явления, преимущественно легкой или средней степени тяжести: наиболее распространенным проявлением являлся запор, возникая у 10–13% пациентов при пероральном приеме семаглутида, у 6% — плацебо и у 19% — при приеме лираглутида 0,9 мг [44]. В исследовании PIONEER-9 пероральный семаглутид более выраженно влиял на гликемический контроль у японских пациентов, чем в международном исследовании PIONEER-1 в глобальной популяции, в котором также оценивалась эффективность пероральной монотерапии семаглутидом. В исследовании PIONEER-1 в глобальной популяции к 26-й неделе терапии пероральным семаглутидом с поправкой на плацебо среднее изменение HbA1c составило в дозе 3 мг -0,6%, в дозе 7 мг -0,9% и в дозе 14 мг -1,1%; в PIONEER-9 — в дозе 3 мг -1,1%, 7 мг -1,5%, 14 мг -1,7% соответственно (рис. 4) [44][45]. Таким образом, японские пациенты могут быть более восприимчивы к гликемическому эффекту агонистов рецептора ГПП-1, чем другие популяции [44].

**Figure:**
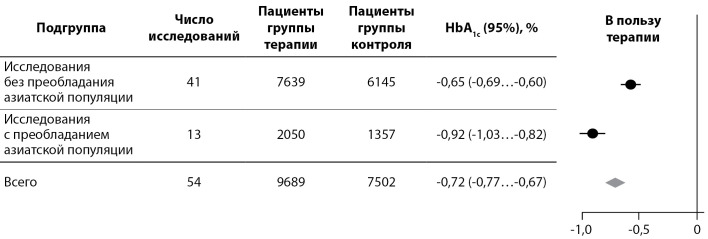
Рисунок 1. Снижение уровня HbA1c на терапии иДПП-4 в исследованиях с >50% или <50% представленностью пациентов из Азии.

**Table:** Таблица 1. Подгрупповой анализ риска MACE в зависимости от расы

Раса	Исследование	Группа терапии арГПП-1	Плацебо	ОР	95% ДИ
Cобытий	Всего	Частота, %	Cобытий	Всего	Частота, %
Европеоидная	All	1,270	10,554	12	1,373	10,595	13	0,92	0,84–1,01
LEADER	494	3,616	13,7	543	3,622	15	0,91	0,81–1,03
SUSTAIN-6	93	1,384	6,7	118	1,352	8,7	0,77	0,59–1,01
EXSCEL	683	5,554	12,3	712	5,621	12,7	0,97	0,87–1,08
Негроидная	All	95	920	10,3	128	956	13,4	0,78	0,60–0,99
LEADER	47	370	12,7	59	407	14,5	0,88	0,60–1,29
SUSTAIN-6	5	108	4,6	7	113	6,2	0,75	0,24–2,35
EXSCEL	43	442	9,7	62	436	14,2	0,68	0,46–1,01
Азиатская	All	72	1,281	5,6	147	1,344	10,9	0,35	0,09–1,32
LEADER	4	435	0,9	56	465	12,0	0,08	0,03–0,21
SUSTAIN-6	8	121	6,6	17	152	11,2	0,59	0,26–1,37
EXSCEL	60	725	8,3	74	727	10,2	0,81	0,58–1,14
Другая	All	82	879	9,3	96	819	11,7	0,78	0,59–1,03
LEADER	27	211	12,8	36	178	20,2	0,63	0,38–1,04
SUSTAIN-6	2	35	5,7	4	32	12,5	0,46	0,08–2,50
EXSCEL	53	633	8,4	56	609	9,2	0,91	0,63–1,33

**Figure:**
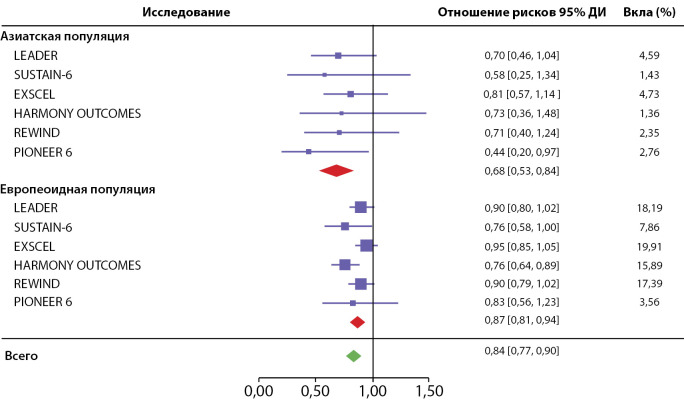
Рисунок 2. Диаграмма отношения риска MACE в различных CVOT-исследованиях арГПП-1 в зависимости от расы.

**Figure:**
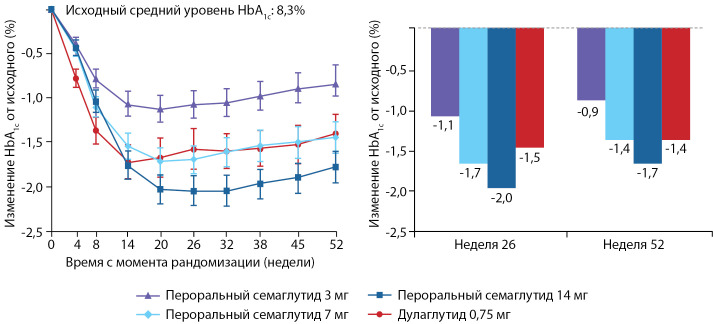
Рисунок 3. Динамика HbA1c в подгруппах применения перорального семаглутида и дулаглутида в исследовании PIONEER-10.

**Figure:**
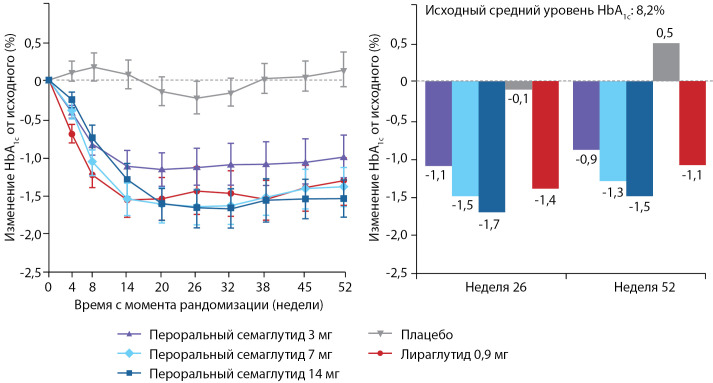
Рисунок 4. Динамика HbA1c в подгруппах применения монотерапии пероральным семаглутидом и лираглутидом в исследовании PIONEER-9.

## ЗАКЛЮЧЕНИЕ

Анализ литературы свидетельствует, что имеются этнические различия в уровне интактного и стимулированного ГПП-1 в азиатской и неазиатской популяции. Вероятно, более низкий уровень ГПП-1 у азиатов может играть значимую роль в развитии и течении СД2, усугубляя дисфункцию β-клеток как ведущего патогенетического звена. Результаты крупных исследований эффективности арГПП-1, перорального семаглутида в частности, показывают достижение лучшего гликемического контроля в группах с преобладанием азиатов. Благоприятная тенденция сердечно-сосудистых исходов в отношении азиатской популяции, несомненно, должна быть подтверждена будущими специально спланированными исследованиями с учетом этнической принадлежности пациентов. Имеющиеся на сегодняшний день результаты исследований арГПП-1 длительного действия позволяют предположить, что эта группа препаратов может быть предпочтительным выбором для азиатских пациентов с СД2, особенно с установленными сердечно-сосудистыми заболеваниями в условиях реальной клинической практики.

## ДОПОЛНИТЕЛЬНАЯ ИНФОРМАЦИЯ

Источники финансирования. Работа выполнена по инициативе авторов без привлечения финансирования.

Конфликт интересов. Хамнуева Л.Ю., Андреева Л.С. получали вознаграждение за участие в научных докладах компании «Ново Нордиск» и других фармацевтических компаний. Подготовка статьи выполнена при поддержке компании «Ново Нордиск».

Участие авторов Хамнуева Л.Ю. — главный (существенный) вклад в концепцию работы, получение, анализ данных, интерпретацию результатов; написание статьи; одобрение финальной версии рукописи; согласие нести ответственность за все аспекты работы, подразумевающую надлежащее изучение и решение вопросов, связанных с точностью или добросовестностью любой части работы; Андреева Л.С. — анализ данных, интерпретация результатов; внесение в рукопись существенной правки с целью повышения научной ценности статьи; одобрение финальной версии рукописи; согласие нести ответственность за все аспекты работы, подразумевающую надлежащее изучение и решение вопросов, связанных с точностью или добросовестностью любой части работы. Все авторы одобрили финальную версию статьи перед публикацией, выразили согласие нести ответственность за все аспекты работы, подразумевающую надлежащее изучение и решение вопросов, связанных с точностью или добросовестностью любой части работы.
